# Neurocysticercosis as a Cause of Epilepsy and Seizures in Two Community-Based Studies in a Cysticercosis-Endemic Region in Peru

**DOI:** 10.1371/journal.pntd.0002692

**Published:** 2014-02-13

**Authors:** Luz M. Moyano, Mayuko Saito, Silvia M. Montano, Guillermo Gonzalvez, Sandra Olaya, Viterbo Ayvar, Isidro González, Luis Larrauri, Victor C. W. Tsang, Fernando Llanos, Silvia Rodríguez, Armando E. Gonzalez, Robert H. Gilman, Hector H. Garcia, for The Cysticercosis Working Group in Peru

**Affiliations:** 1 Cysticercosis Elimination Program and Center for Global Health Tumbes, Universidad Peruana Cayetano Heredia, Tumbes, Perú; 2 Department of International Health, Bloomberg School of Public Health, Johns Hopkins University, Baltimore, Maryland, United States of America; 3 U.S. Naval Medical Research Unit No. 6, Lima, Perú; 4 Instituto de Ciencias Neurológicas, Lima, Perú; 5 Georgia State University, Atlanta, Georgia, United States of America; 6 School of Public Health, Universidad Peruana Cayetano Heredia, Lima, Perú; 7 School of Veterinary Medicine, Universidad Nacional Mayor de San Marcos, Lima, Perú; 8 Department of Microbiology, School of Sciences, Universidad Peruana Cayetano Heredia, Lima, Perú; Universidad Nacional Autónoma de México, Mexico

## Abstract

**Background:**

The prevalence of epilepsy added to inadequate treatment results in chronic morbidity and considerable mortality in poor populations. Neurocysticercosis (NCC), a helminthic disease of the central nervous system, is a leading cause of seizures and epilepsy in most of the world.

**Methods:**

Taking advantage of a cysticercosis elimination program, we performed two community-based cross-sectional studies between 2006 and 2007 in 58 rural communities (population 20,610) to assess the prevalence and characteristics of epilepsy and epileptic seizures in this endemic region. Serological and computed tomography (CT) data in individuals with epilepsy were compared to previous surveys in general population from the same region.

**Principal findings:**

In two surveys, 17,450 individuals were evaluated. Lifetime prevalence of epilepsy was 17.25/1000, and prevalence of active epilepsy was 10.8/1000 inhabitants. The prevalence of epilepsy increased after age 25 years and dropped after age 45. Only 24% (45/188) of patients with active epilepsy were taking antiepileptic drugs, all at sub-therapeutic doses. Antibodies to cysticercosis were found in approximately 40% of individuals with epilepsy in both studies. In one survey only individuals presenting strong antibody reactions were significantly associated with having epilepsy (OR 5.74; p<0.001). In the second, the seroprevalence as well as the proportion presenting strong antibody reactions were both significantly higher in individuals with epilepsy (OR 2.2 and 4.33, respectively). Brain CT showed NCC-compatible images in 109/282 individuals with epilepsy (39%). All individuals with viable parasites on CT were seropositive.

**Conclusion:**

The prevalence of epilepsy in this cysticercosis endemic region is high and NCC is an important contributor to it.

## Author Summary

Epilepsy is a chronic disease affecting more than 50 million people around the world. In many countries neurocysticercosis (NCC), a parasitic disease of the central nervous system, is a frequent cause of seizures and epilepsy. After a human ingests poorly cooked pork with cysts of the tapeworm *Taenia solium*, the larvae develops in the small intestine as an adult tapeworm. The tapeworm carrier becomes a source of infective eggs which are released with the stools and can infect humans or pigs with the larval stage, causing cysticercosis. There are many forms of clinical manifestations of NCC; epilepsy is one of them and the most frequent. Scarce population based-studies to identify individuals with epilepsy in rural communities exist, including only a few from Latin America. Our community-based study used a validated survey applied by trained non-medical professionals (nurse, nurse technicians, mid-wife, etc.) and found 301 individuals with epilepsy (17.25/1000), 70% of them without antiepileptic medication and 39% of them with images suggestive of NCC. The prevalence of epilepsy is high in this region and cysticercosis is a frequent finding in individuals with epilepsy.

## Introduction

Epilepsy is a neurological disorder characterized by sudden, recurrent and unpredictable interruptions of normal brain function [1]-[4]. This condition results in chronic morbidity and considerable mortality in resource-poor populations because of its high prevalence and inadequate and late treatment [3]–[10].

One of the leading causes of seizures and epilepsy in developing countries is neurocysticercosis (NCC), a helminthic disease of the central nervous system [5], [7]–[9]. Trauma, genetic predisposition, other infections and social and cultural factors contribute to epilepsy prevalence worldwide [5]. Available epidemiologic data on risk factors for epilepsy is mostly based on North American or European studies, with only a few studies from India, Latin American, or African countries [5], [11], [12]. It is to be expected that etiology, age-specific data and other characteristics will be different in poor regions [13]. Population-based estimates of the contribution of NCC to the prevalence of seizures and epilepsy in endemic regions have been published before, in most cases within a single community or with limited sample size [3], [5], [8], [9], [14].

In a smaller study in Matapalo, Peru (n = 903), we previously reported the prevalence of active epilepsy to be 16.6 per 1000, with 39% of individuals with epilepsy having evidence of NCC on CT [8]. Taking advantage of a large cysticercosis elimination program, we used a similar study design in two community-based cross-sectional studies to better estimate the prevalence and characteristics of epilepsy and epileptic seizures in this cysticercosis-endemic region, as well as to examine the contribution of NCC to the seizure burden.

## Methods

### Study site

**Figure 1:**
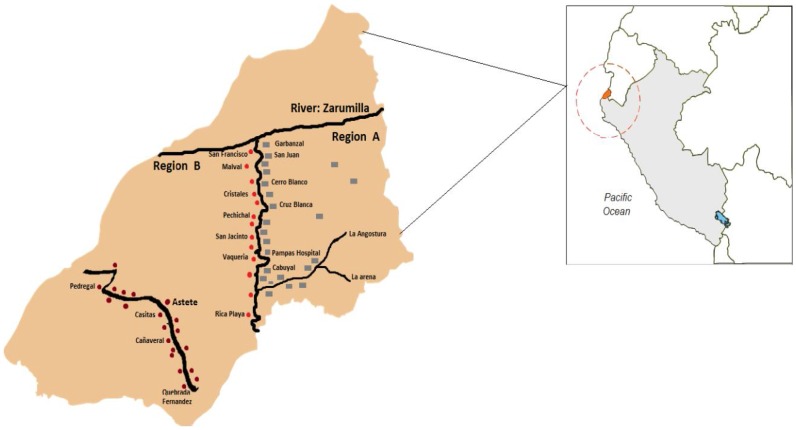
Map of Tumbes and rural communities intervened in 2006 (region A) and 2007 (region B).

We conducted two cross-sectional studies, involving 20,610 individuals from 58 communities of the Northern coast of Peru. The first study was conducted in 2006 on the right bank of the Tumbes River (Region A), and the second was conducted in 2007 on the left bank of the same river (Region B) (Figure 1). No significant changes in sanitation or other conditions occurred in this rural area between the first and second studies. The study area, with an extension of 4669.2 km^2^, is known to be endemic for cysticercosis and has a high prevalence of epilepsy (up to 32.1 per 1000 inhabitants) [7]. The population in the area is mostly Mestizo, a mixture of Spaniard and Amerindian. Rice and banana cultivation are the largest economical activities followed by coastal fishery. Most villages have electricity but not sewage facilities nor running water. There are 19 basic-level health centers in both study areas; each staffed by a recently graduated physician performing their one-year rural service, a nurse and health technicians.

### Enrollment and evaluations

The study was presented to the local health authorities and community leaders of each of the study villages to ensure their acceptance and collaboration, and then activities were performed in three phases:

**Phase I - Epilepsy screening survey.** A door-to-door baseline census was carried out to obtain basic demographic information. After appropriate informed consent, a nine-question survey to identify epilepsy-related neurological symptoms was administered by laboratory technicians, nurses and obstetric technicians previously trained by a neurologist and two medical supervisors. This survey tool was originally developed by Placencia [10] then modified and validated by the same team [15] and others [5]. Training involved educational material, lectures, and practice sessions for approximately a week, plus an online course on human subject research protection.

**Phase II - Medical evaluation.** Consenting positive respondents to the survey were interviewed and evaluated in the local health centers by two physicians to further define suspected cases of seizures or epilepsy, or to exclude other disorders or symptoms. These general practitioners were previously trained to recognize epileptic and non-epileptic seizures and had also participated in similar studies before. Particular care was taken to include local words like “*alferecia*”, “*susto*”, “*espanto de los encantos*” or "*daño*" which are used by the local rural population to describe seizures.

**Figure 2:**
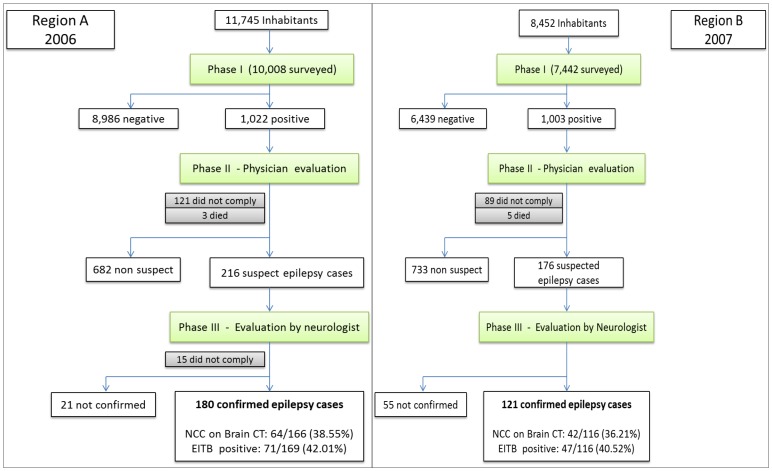
Study Flowchart for region A (2006) and region B (2007).

**Phase III - Neurological examination, blood sampling and brain CT scan.** A team of certified neurologists evaluated suspected epilepsy cases for case confirmation and to rule out non-epileptic events. Seizures were classified as being of partial or generalized onset and the diagnoses of epileptic seizures or epilepsy were confirmed according to the criteria and definitions of the International League against Epilepsy (Figure 2, Study Flowcharts).

Confirmed cases had a blood sample taken for enzyme-linked immunoelectrotransfer blot (EITB) for cysticercosis using lentil-lectin, affinity–purified *Taenia solium* metacestode glycoprotein antigens [16], [17]. Serology has been widely used as a proxy for cysticercosis infection or disease in endemic communities as brain imaging is rarely available in these poor settings. In endemic communities, only a minority of antibody-positive individuals have epilepsy.[5], [8], [9] In addition, approximately half of those individuals with neurocysticercosis and epilepsy present calcified cysts only and their serology has already converted to negative.[8] Non-contrasted brain computed tomography (CT) scan was offered to individuals with a confirmed diagnosis of epilepsy or seizures and performed using a helicoid CT scan (Siemens AG, Germany) in the facilities of the Cysticercosis Elimination Program in Tumbes. Women in reproductive age had a urine pregnancy test performed before brain CT scan.

### Comparison data

We used archived serologic data (EITB) from a previous population-based study in 14 of the 58 study communities to estimate the background seroprevalence. In addition, we used 111 brain CT scans taken in a previous study in Tumbes from individuals without a history of epilepsy to estimate the prevalence of NCC in the general population [8]. The studies were conducted between 2005 and 2007.

### Study Definitions

**Resident.** A person who had slept in the village two or more days per week on average during the last three months [8].

**Epileptic seizure.** Clinical manifestation presumed to result from an abnormal and excessive discharge of a set of neurons in the brain, perceived by the patient or an observer (include alteration of consciousness or motor, sensory, autonomic or psychic events) [8], [11], [18], [19].

**Epilepsy.** Two or more unprovoked seizures in a period of more than 24 hours. Multiple seizures occurring in a 24-hour period are considered a single event [8], [11], [18], [19].

**Active epilepsy.** At least one epileptic seizure in the previous five years, regardless of antiepileptic drug (AED) treatment [8], [18].

**Non-active epilepsy.** Remission with treatment (a person with epilepsy who had no seizures in the last five years and was receiving treatment at the time of ascertainment) or remission without treatment (a person with epilepsy who had no seizures in the last five years and was not receiving treatment at the time of ascertainment) [15], [20].

**Lesions compatible with NCC on CT scan.** Single or multiple cystic, degenerating or calcified lesions in the brain parenchyma, with or without edema, or extraparenchimal lesions (subarachnoid or intraventricular cysts) [21], [22].

**Blood sampling (serology).** Blood samples (5 cc) were taken from all consenting participants by venipuncture.

**Positive EITB.** One or more reactive specific antibody bands in serum samples assayed by EITB for cysticercosis. Reactions to four or more reactive specific antibody bands in serums assayed by EITB for cysticercosis were defined as strong antibody reactions and analyzed separately.

### Ethical considerations

The study protocol and consent forms were reviewed and approved by the institutional review boards of the Universidad Peruana Cayetano Heredia and the Johns Hopkins University Bloomberg School of Public Health. Appropriate informed consent procedures were followed including a signed informed consent form obtained in adults (more than 18 years old) and an assent form for individuals younger than 18 years (parents/guardians also signed an additional written informed consent form).

### Statistical analysis

Chi square test and Fisher’s exact test were used to compare associations between categorical variables. Lifetime prevalence was defined as the number of persons with seizures or epilepsy detected divided by the number of respondents to the baseline survey. The 2007 Peru national census was used as reference population for age-adjustments in prevalence. Confidence intervals for prevalence estimates were estimated based on exact binomial method. Odds ratios (OR) were estimated in univariate logistic regression, and adjusted OR (aOR) were estimated in a multiple logistic regression. All reported probability (p) values were two-sided with a significance level set at 0.05. Statistical analyses were carried out using Stata version 11.1 (Stata Corp., College Station, TX, USA).

### Limitations

Magnetic resonance imaging (MRI) and EEG are not available on this area. Non-contrast brain CT scan as performed in this study is a useful tool to identify cysts and calcified cysticercosis lesions but small enhancing lesions without edema could have been missed.

## Results

### Study population

The first study, in 45 communities of the right bank of the Tumbes River and surrounding areas, was performed between April 2006 and January 2007. The second study involved 13 larger communities of the left bank of the Tumbes River and was performed between January and October 2007. From a total combined base population of 20,610 censused inhabitants, those aged 2 years or older (n = 20,197) were invited to participate in the door to door surveys and 17,452 (86.41%) consented to participate. Villages in the second study were larger and the participants were older (32 versus 27 years, p<0.001, Table 1). Coverage and sex distribution were quite similar between studies. Both study populations were rural and poor. A large proportion of the population surveyed in 2006 had access to in-house potable water, and a larger proportion reported open defecation.

**Table 1:** Demographic characteristics of two wide scale cross-sectional studies in 2006 and 2007.

variable	Region A (2006)	95% CI	Region B (2007)	95% CI
	(%)	(%)	(%)	(%)
Base population	11,935		8,675	
	(11,745 age 2 or older)		(8,452 age 2 or older)	
Communities	45		13	
Consenting participants	10,008		7,442	
Coverage	85.21	83.19– 84.51	88.05	87.35– 88.74
Households	2,882		2,157	
Male	5,180 (51.76)	50.77– 52.73	4,361 (51.60)	50.53– 52.66
Median age	27 (SD 20.08)		32.23 (SD 21.10)	
In-house potable water	5,074 (43.20)	42.30– 44.09	2,208 (26.12)	25.18– 60.48
Water from river or wells	5,191 (44.20)	43.29– 45.09	5,200 (61.52)	60.48– 62.56
Defecate in the field	3,698 (31.49)	30.64– 32.32	1,371 (16.22)	15.43– 17.00
Raised pigs	6,960 (59.26)	58.37– 60.14	4,547 (53.80)	52.73–54.86

### Differences between participants and non-respondents

Compared with survey participants, individuals who did not respond to the survey were more frequently male (1,022/1,737, 58.84% in 2006, and 630/1,010, 62.37% in 2007, p<0.001 in both surveys). There were no differences in age or most common sources of water - except for buying from water trucks, which was more frequent in non-respondents in the 2006 study, and less frequent in the 2007 study. Other differences were present in one survey but not in the other (data not shown).

### Epilepsy survey

The proportions of positive respondents to the epilepsy questionnaire were 10.21% in 2006 (1,022/10,008) and 13.47% in 2007 (1,003/7,442). In 2006, 124 individuals did not have a medical examination due to diverse reasons. In 2007, 99 individuals did not have a medical/neurological examination. The lifetime prevalence of epilepsy was 17.25/1000 and that of active epilepsy was 10.8/1000 inhabitants, without marked differences between surveys (table 2). After direct age-adjustment to the 2007 Peruvian national census, lifetime epilepsy prevalence was 16.61/1000 and active epilepsy was 10.39/1000 inhabitants.

**Table 2:** Prevalence’s of epilepsy and seizures in the studied populations.

variable	Region A (2006)	95% CI	Region B (2007)	95% CI	Combined	95% CI
	prevalence	%	prevalence	%		%
Consenting participants	10,008		7,442		17,452	
Positive survey	1,022		1,003		2,025	
	10.21%		13.47%		11.60%	
Confirmed epilepsy cases	180 (18/1000)	15.5 to 20.8	121 (16.3/1000)	13.5 to 19.3	301 (17.25/1000)	17.1 to 21.4
Active epilepsy	107 (10.7/1000)	8.7 to 12.9	81 (10.9/1000)	8.6 to 13.5	188 (10.8/1000)	9.3 to 12.4
Non-active epilepsy	73 (7.3/1000)	5.7 to 9.2	40 (5.3/1000)	3.8 to 7.3	113 (6.5/1000)	5.3 to 7.8
Single seizure	68 (6.8/1000)	5.2 to 8.6	40 (5.3/1000)	3.8 to 7.3	108 (6.2/1000)	5.1 to 7.5

Individuals diagnosed as having had febrile seizures only (n = 32 and n = 20 in 2006 and 2007 respectively) were not included [3], [8]. Individuals with epilepsy were older than negative survey respondents (mean age 34 ± 18.69 versus 27 ± 21.01 years; p = 0.036 in 2006; mean age 36.1 ± 18.6 versus 31.7 ± 21.1; p = 0.033 in 2007) but there were no differences in regard to sex, stool disposal, proportion which raised pigs, or mean numbers of pigs raised (data not shown).

**Figure 3:**
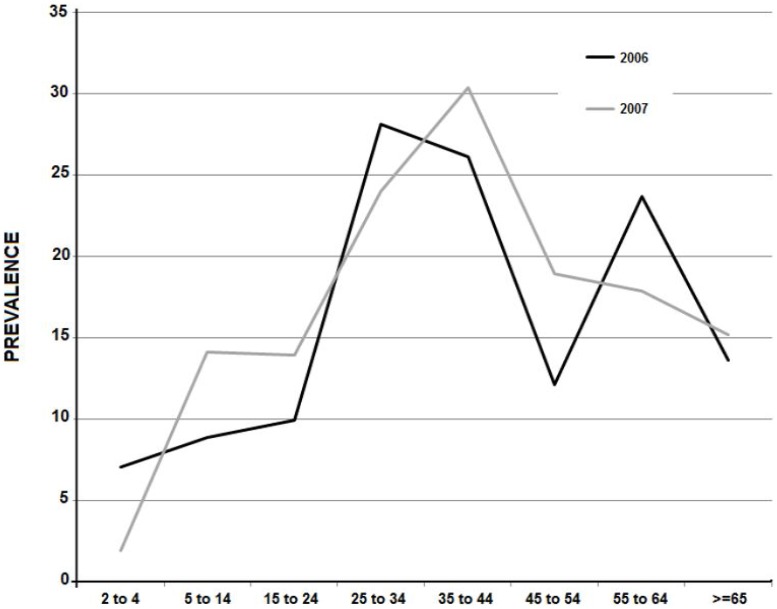
Prevalence of epilepsy stratified by age in 58 rural communities during the population based-studies interventions in 2006 (region A) and 2007 (region B).

Seizures were classified as partial or of partial onset in 149 cases, generalized in 126 cases, and undefined in 26. There were no significant differences between the 2006 and 2007 surveys in regard to the proportion of patients with generalized seizures (70/180, 38.89%, versus 56/121, 46.28%; p = 0.202). Partial seizures were more frequently reported in 2006 (99/180, 55%, versus 50/121, 41.32%; p = 0.020), and undefined seizures were more frequently reported in 2007 (11/180, 6.11%, versus 15/121, 12.3%; p = 0.057). The age at first seizure were quite similar in both surveys and were as follows: before age 5 in 98 cases (32.77%), 28 of them before one year-old; between 6 and 10 years in 60 (20.06%); between 11 and 15 years in 48 (16.05%); between 16 and 20 years in 27 (9.03%), and older than 20 years in 66 (22.09%). The prevalence of epilepsy by age increased after age 25 years and dropped after age 45 in both the 2006 and the 2007 surveys (Figure 3).

We found a huge epilepsy treatment gap in this developing region. While most of the individuals with active epilepsy had previously received medical care at the local health center at some point, at the time of this study only 45 out of 188 (23.94%) patients with active epilepsy (30/107 and 15/81 from 2006 and 2007 respectively) were taking AEDs. All of them were receiving sub-therapeutic doses, either 100 mg/d phenytoin or 200 mg/d carbamazepine, and none of them had obtained seizure remission. In most cases the prescription had been received from non-medical staff or even from a witch doctor (commonly prescribing sub-therapeutic AED doses mixed with a “cured bottle”, an extract of medicinal herbs in cane or grape alcohol).

### Antibody seroprevalence in individuals with epilepsy and neurologically asymptomatic individuals

The seroprevalence in individuals with epilepsy was approximately 40% in both studies, but the background reference seroprevalence differed between the communities studied in 2006 and those studied in 2007. The analyses of the associations between antibody responses and epilepsy for each study are thus presented separately below.

**Serological results in Region A, 2006.** The serological results of individuals with epilepsy (169 consenting out of 180) were compared to those of neurologically asymptomatic general population from seven of the same study villages, previously collected as part of a different study. Both groups were similar in age or sex distribution. Compared to the individuals with epilepsy, the comparison group from general population were more likely to defecate in the field (49/169, 28.99%, versus 83/969, 8.65%, p<0.001), raise pigs (657/959, 68.51%, versus 102/169, 60.36%, p = 0.029), or obtain water from the river (89/169, 52.66%, versus 445/959, 46.40%, p = 0.045).

The seroprevalence in general population was 35.87% (344/959), with 16 individuals (1.5%) having strong serological reactions (4 to 7 bands). The seroprevalence in individuals with epilepsy was 42.01% (71/169), not statistically different from that of the comparison general population group. However, the proportion presenting strong antibody reactions was much higher in individuals with epilepsy (15/169, 8.87% versus 16/959, 1.65%, OR 5.74; 95%CI: 2.8–11.9, p<0.001).

**Serological results in Region B, 2007.** The serological results of individuals with epilepsy (116 consenting out of 121) were compared to those of neurologically asymptomatic general population from seven of the same study villages, previously collected as part of a different study. Compared to the individuals with epilepsy, the comparison group from general population was younger (33.1 +/– 21.48 years versus 37.2 +/– 18.08 years, p = 0.043), but both groups were similar in distribution by sex, source of water, stool disposal, type of household, and frequency of pig rising.

The seroprevalence in general population was 23.4% (656/2808), with 22 individuals (0.78%) having strong serological reactions (4 to 7 bands). The seroprevalence in individuals with epilepsy was 40.5% (47/116), significantly higher (OR 2.2; 95% CI 1.52 to 3.27, p<0.001). The proportion presenting strong antibody reactions was also higher in the epilepsy group (4/121, 3.31% versus 22/2,808, 0.78%, OR 4.33; 95% CI 1.5 to 12.7 p = 0.004).

### Brain CT scan

Most (282 out of 301) individuals with epilepsy had a brain (CT) scan performed. CTs showed NCC-compatible images in 109 (39%). These individuals had viable intraparenchymal cysts in six cases (cysts as the only finding in two; median number was 5.09 cysts, range from 1 to 36), subarachnoid cysts in three cases (always associated with other findings), calcifications in 100 cases (calcifications as only finding in 91; median number of calcifications was 3.57, range from 1 to 67), and hydrocephalus in 11 cases (hydrocephalus as only finding in six). Combinations of CT findings included viable cysts and calcifications (n = 3), subarachnoid cyst with calcifications (n = 2), hydrocephalus and calcifications (n = 4), or viable cysts, subarachnoid NCC and hydrocephalus (n = 1).

When comparing CT findings in this series to the corresponding control groups from our Matapalo study [8], the overall proportion of CT-positive findings was significantly higher than that reported in non-epileptic general population of similar villages (109/282 [39%] vs. 26/111[23%] ; OR: 2.05; 95% CI: 1.25–3.39,p =  0.004). There were no significant differences in terms of proportion with NCC between individuals with active or inactive epilepsies (66/185 [35.68%] vs. 43/97 [44.33%]; p = 0.156), nor between those with partial seizures and those with generalized seizures.

### Brain CT and serology

There were 279 individuals with epilepsy who had both a brain CT scan and EITB serology performed, of which 115 were seropositive (41.21%). NCC compatible images were present in 62/115 (53.91%) of the seropositive and 47/164 (33.56%) of the seronegative (OR 2.82, 95% IC 1.72 to 4.65, p = <0.001). All individuals with viable parasites on CT were seropositive (Table 3). From 91 individuals with calcifications as their only CT finding, multiple calcifications (more than 2) were more frequent in those seropositive compared to seronegative individuals (33/48, 68.75%, versus 19/43, 44.18%, p = 0.018).

**Table 3:** NCC-compatible CT findings in seronegative and seropositive individuals with epilepsy in Tumbes, Peru.

NCC-compatible CT	Seropositive	Seronegative
(n = 109)	(n = 62)*	(n = 47)
Subarachnoid NCC, with or without other lesions	3	0
Viable cysts, with or without edema	6	0
Calcified cysts without hydrocephalus	53	43
Calcified cysts with hydrocephalus	2	2
Hydrocephalus only	4	2

* Some patients had more than one finding.

In subgroup analysis, 60% (62/115) of the seropositive individuals with epilepsy had evidence of NCC on CT, compared to 35% (18/53) previously found in villagers without epilepsy and seropositive [8] (OR 2.27, 95%CI 1.15 to 4.47, p = 0.016); similarly 29% (48/164) of the seronegative individuals with epilepsy had evidence of NCC on CT, compared to 14% (8/58) of villagers without epilepsy and seronegative [8] (OR 2.58, 95% CI 1.14 to 5.86, p = 0.02).

## Discussion

This population based-study in rural communities found a lifetime epilepsy prevalence of 17.2/1,000 (301/17,450), and a prevalence of active epilepsy of 10.8/1,000 (188/17,450). These values are similar or slightly lower than others obtained in rural Latin-America studies (lifetime prevalence 17.8/1000; active epilepsy 12.4/1000) [3], [5], [6], [9]. The usual prevalence of epilepsy in non-endemic industrialized countries ranges between 2.7 and 7.1 per thousand inhabitants [23], [24]. The Center for Disease Control and Preventiońs Behavioral Risk Factor Surveillance System (BRFSS), estimates the prevalence of active epilepsy in the US to be 8.4/1000 and the lifetime prevalence of epilepsy to be 16.5/1000 [11], although its results may be increased by the use of a self-reporting system. Our values are likely underestimated since 223 out of 2025 (11%) positive respondents to the epilepsy survey did not comply with the medical or neurological interview. Simple adjustment including a similar proportion of those survey positive respondents who did not attend the clinical examination would take the prevalence of epilepsy to 19.2/1,000, and that of active epilepsy to 11.9/1,000.

Our data also showed an important contribution of NCC to epilepsy cases. Thirty-nine percent of our cases had NCC-compatible images on CT, above the expected values for the general population. There were 15 individuals with epilepsy who had viable brain cysts, subarachnoid NCC, or hydrocephalus, all conditions which are not expected to be found in comparison individuals without neurological symptoms. Also, individuals with epilepsy and only calcified NCC had multiple calcifications (median 3.6), rather than the more frequent finding of a single parenchymal brain calcification in endemic regions. In most community-based studies using similar methodology, brain CT scans were only taken in individuals with active epilepsy [3], [5], [9], [25]. In our study NCC was present in 35.1% of individuals with active epilepsy, a proportion similar to those found in Salama, Honduras (36.6%, n = 6473 residents) [3] and Vellore, India (34.0%, n = 50 617 individuals) [24], and slightly higher than in rural Ecuador (26.3%, n = 2415 residents) [5] and rural Bolivia (27, 4%, n = 10,124) [9].

Seropositivity was also highly prevalent in individuals with epilepsy, and was associated with viable NCC as well as increased numbers of lesions. Of particular interest, a strong antibody response was associated with a higher risk of epilepsy in both surveys, despite the high background seroprevalence in the villages surveyed in 2006.

Not surprisingly, our participants share the fate of most individuals with epilepsy in developing countries, where the lack of neurology in rural communities and lack of appropriate treatment in individuals with epilepsy worsen the prognosis [23], [24]. Most patients were not receiving AED therapy. Those that were receiving it did not have the drug prescribed by a doctor. Instead, the drug was provided by a Shaman in sub-therapeutic doses. The most frequent form of therapy referred to by our patients was called "cure bottle" (a bottle of liquid made by a Shaman from hard liquor and plants prescribed twice a day). Sometimes Shamans added an AED like carbamazepine or phenobarbital temporarily to the bottle, in periods of increased seizure frequency or according to the phases of the moon. The epilepsy treatment gap in this rural region (seventy-six percent) is even higher than recently reported estimates from rural Africa where it was 62% [26].

Our data may be affected by unaccounted biases or external effects. The use of CT instead of MRI may have resulted in misclassification by missing individuals with small cysts, cysts in the ventricles, or cysts close to the skull, thus diluting the association effect. Unfortunately, MRI was not available in the area as is typical of cysticercosis-endemic regions. On the other hand, NCC may have coexisted with seizures from other etiologies in a subset of individuals, increasing the effect size. Despite the consistency of the association between antibody serology and seizure disorders as shown in this and other studies, still most seropositive individuals in field conditions do not seem to have neurological symptoms, and a significant proportion of symptomatic individuals with NCC have already calcified disease and their antibody serology has converted to negative. It follows that the use of serology in general population studies would give only a gross estimation of the magnitude of transmission rather than a precise detection of clinical cases.

Our data provides an estimate for epilepsy prevalence in rural Latin America and largely confirms previous surveys, most of them with small size, in the sense that prevalences in this region are higher than those in the US or Europe. There was an almost complete lack of adequate seizure management in the first level of care, exposing an evident deficiency which should be targeted by the local health authorities. It also supports the idea that NCC is a strong contributor to seizures and epilepsy in endemic regions. This population-based study was performed simultaneously with a large scale cysticercosis elimination program. If cysticercosis elimination is obtained, correlation of the decreases in cysticercosis transmission with the trends in seizure incidence in the following years may open the possibility of avoiding close to 30% of cases of seizures and epilepsy in vast regions of the world.

## Supporting Information

Checklist S1 STROBE Checklist.
(PDF)
